# Computed tomography evaluation of the morphometry and variations of the infraorbital canal relating to endoscopic surgery^☆^

**DOI:** 10.1016/j.bjorl.2017.08.009

**Published:** 2017-09-08

**Authors:** Gülay Açar, Kemal Emre Özen, İbrahim Güler, Mustafa Büyükmumcu

**Affiliations:** a Necmettin Erbakan University, Meram Faculty of Medicine, Department of Anatomy, Konya, Turkey; b İzmir Kâtip Çelebi University, Faculty of Medicine, Department of Anatomy, İzmir, Turkey; c Selcuk University, Faculty of Medicine, Department of Radiology, Konya, Turkey

**Keywords:** Endoscopic sinus surgery, Infraorbital canal, Infraorbital canal corpus types, Infraorbital foramen, Multidetector computed tomography, Cirurgia endoscópica sinusal, Canal infraorbitário, Tipos de corpo do canal infraorbitário, Forame infraorbitário, Tomografia computadorizada multidetectores

## Abstract

**Introduction:**

The course of the infraorbital canal may leave the infraorbital nerve susceptible to injury during reconstructive and endoscopic surgery, particularly when surgically manipulating the roof of the maxillary sinus.

**Objective:**

We investigated both the morphometry and variations of the infraorbital canal with the aim to show the relationship between them relative to endoscopic approaches.

**Methods:**

This retrospective study was performed on paranasal multidetector computed tomography images of 200 patients.

**Results:**

The infraorbital canal corpus types were categorized as Type 1: within the maxillary bony roof (55.3%), Type 2: partially protruding into maxillary sinus (26.7%), Type 3: within the maxillary sinus (9.5%), Type 4: located anatomically at the outer limit of the zygomatic recess of the maxillary bone (8.5%). The internal angulation and the length of the infraorbital canal, the infraorbital foramen entry angles and the distances related to the infraorbital foramen localization were measured and their relationships with the infraorbital canal variations were analyzed. We reported that the internal angulations in both sagittal and axial sections were mostly found in infraorbital canal Type 1 and 4 (69.2%, 64.7%) but, there were commonly no angulation in Type 3 (68.4%) (*p* < 0.001). The length of the infraorbital canal and the distances from the infraorbital foramen to the infraorbital rim and piriform aperture was measured as the longest in Type 3 and the smallest in Type 1 (*p* < 0.001). The sagittal infraorbital foramen entry angles were detected significantly smaller in Type 3 and larger in Type 1 than that in other types (*p* = 0.003). The maxillary sinus septa and the Haller cell were observed in 28% and 16% of the images, respectively.

**Conclusion:**

Precise knowledge of the infraorbital canal corpus types and relationship with the morphometry allow surgeons to choose an appropriate surgical approach to avoid iatrogenic infraorbital nerve injury.

## Resumo

**Introdução:**

O trajeto do canal infraorbitário pode predispor o nervo infraorbitário a lesões durante cirurgias reconstrutoras e endoscópicas com manipulação do teto do seio maxilar.

**Objetivo:**

Investigamos a morfometria e as variações do canal infraorbitário e objetivamos demonstrar a relação entre elas, visando as abordagens endoscópicas.

**Método:**

Este estudo retrospectivo foi realizado em imagens de tomografia computadorizada multidetectora de seios paranasais de 200 pacientes.

**Resultados:**

Os tipos de corpos do canal infraorbitário foram categorizados como Tipo 1; inseridos no teto ósseo maxilar (55,3%), Tipo 2; projetando-se parcialmente dentro do seio maxilar (26,7%), Tipo 3; dentro do seio maxilar (9,5%), Tipo 4; localizado anatomicamente no limite externo do recesso zigomático do osso maxilar (8,5%). A angulação interna e o comprimento do canal infraorbitário, os ângulos de entrada do forame infraorbitário e as distâncias relacionadas à localização do forame foram medidos e suas relações com as variações do canal infraorbitário foram analisadas. Observamos que as angulações internas em ambos os cortes sagital e axial foram encontradas em sua maioria em canais infraorbitários Tipo 1 e 4 (69,2%, 64,7%) e, no geral, não houve angulação no canal Tipo 3 (68,4%) (*p* < 0,001). O comprimento do canal infraorbitário e as distâncias desde o forame infraorbitário até o rebordo infraorbitário e a abertura piriforme foram medidos e os mais longos foram identificadas no Tipo 3 e os mais curtos no Tipo 1 (*p* < 0,001). Os ângulos de entrada do forame infraorbitário em projeção sagital foram significativamente menores no Tipo 3 e maiores no Tipo 1, em relação aos outros tipos (*p* = 0,003). Septos nos seios maxilares e as células de Haller foram observados em 28% e 16% das imagens, respectivamente.

**Conclusão:**

O conhecimento preciso dos tipos de corpo do canal infraorbitário e a relação com a morfometria permitem que o cirurgião escolha uma abordagem cirúrgica apropriada para evitar lesões iatrogênicas do nervo infraorbitário.

## Introduction

The maxillary nerve that leaves the skull base through foramen rotundum gives off an infraorbital nerve (ION) in the pterygopalatine fossa. The ION is responsible for sensory innervation to the skin of the face from lower eyelid to upper lip enters through the inferior orbital fissure and courses anteriorly in the infraorbital groove (IOG) over the orbital floor and enters into the infraorbital canal (IOC) which is opened by the infraorbital foramen (IOF) under the infraorbital rim (IOR).^1^ The IOC, which is covered with the extremely thin bone, is one of the weakest points of the orbital floor and thus provides the least support to orbital bony strength. So, orbital floor fractures and surgeries like endoscopic approaches, orbital decompression and reconstruction can cause ION injury, which can result in massive hemorrhage, complete anesthesia or progressive infraorbital hypoesthesia from ION entrapment.^2^

The IOC commonly courses upward and laterally within the maxillary sinus roof but it can protrude from the maxillary corpus into the sinus as seen in sagittal sections of the computed tomography (CT) scans. The increasing degree of the protrusion of the IOC can cause iatrogenic ION injury during surgical operations manipulating or reconstructing the orbital floor.^1^ Therefore, having a precise knowledge of the anatomic variations and the morphometry of the IOC is critical for surgeons. In addition, preoperative radiological evaluation of the IOC corpus types is necessary for surgical management of reconstruction of the orbital floor, regional ION block and radiofrequency ablation neurotomy in V2 trigeminal neuralgia.^3^

In the literature, there are many studies concerning the variations or morphometry of the IOC, IOG and IOF. But, there is no clinical study investigating whether these morphometric parameters are altered by the IOC corpus types. Therefore, it was of great interest to us to study this topic. In this study, we especially focused on the internal angulation of the IOC and aimed to find out whether there are factors that are of value in evaluating the ION injury. We analyzed the relationship between the morphometry and the variations of the IOC corpus types by using CT scans of actual patients and tried to show its potential utility as an endoscopic surgical landmark. Also, we evaluated the surrounding structures such as maxillary sinus septa and Haller cell.

## Methods

This retrospective study was approved by our local ethics committee with a number 2016/522 and performed using paranasal Multidetector Computed Tomography (Syngo Via) images of 200 patients who presented to the Department of Radiology for clinical purposes between January 2015 and December 2015. Axial CT images were obtained with a section thickening of 0.625 mm, and these source data were used to obtain associated coronal and sagittal images of 1 mm slice thickness. No patient underwent a new CT examination for this study. Patients with paranasal sinus anomalies, orbital fractures and patients who had previous sinus surgery were excluded from the study.

We categorized the IOC corpus types according to the relationship with the maxillary sinus into four types as follows, which are based on the Ference et al.^2^ classification of the ION and Rusu et al.^4^ definition of the lateroantral type.

**Figure 1:**
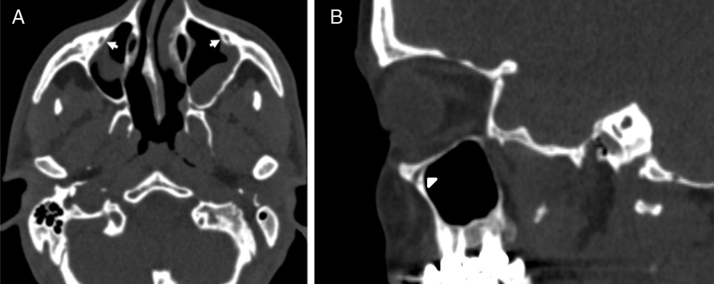
(A) Axial paranasal sinus CT image showing right-sided infraorbital canal Type 1 within the maxillary bony roof and left-sided infraorbital canal Type 2 partially protruding into maxillary sinus (thick arrows). (B) Right parasagittal image showing infraorbital canal Type 1 (arrowhead).
IOC Type 2, partially protruding into the maxillary sinus (Fig. 2A and B).

IOC Type 1, within the maxillary bony roof (Fig. 1A and B).

**Figure 2:**
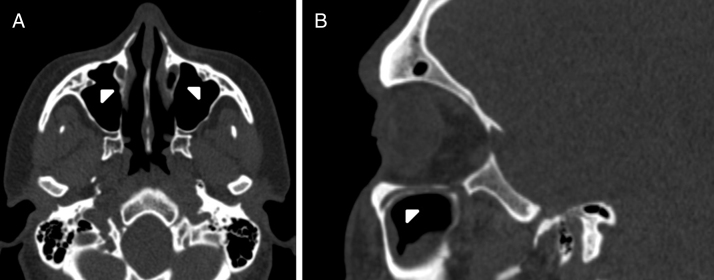
(A) Axial paranasal sinus CT image showing bilateral infraorbital canal Type 2 partially protruding into the maxillary sinus (arrowheads). (B) Right parasagittal image showing infraorbital canal Type 2 (arrowhead).
IOC Type 3, totally protruding into the maxillary sinus with a stalk (Fig. 3A and B).

**Figure 3:**
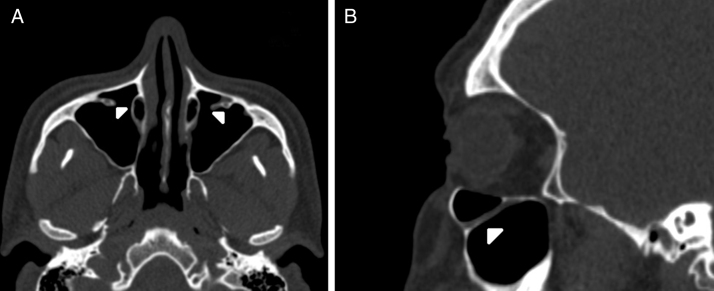
(A) Axial paranasal sinus CT image showing bilateral infraorbital canal Type 3 totally protruding into the maxillary sinus with a stalk (arrowheads). (B) Right parasagittal image showing infraorbital canal Type 3 (arrowhead).
IOC Type 4, located anatomically at the outer limit of the zygomatic recess of the maxillary bone (lateroantral) (Fig. 4A and B).

**Figure 4:**
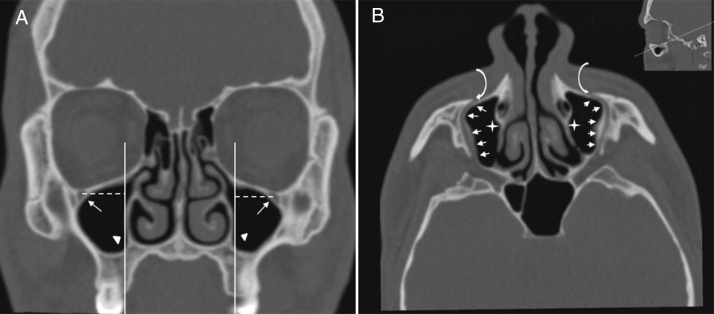
(A) Coronal paranasal sinus CT image showing bilateral infraorbital canal Type 4 which is located at the outer limit of the zygomatic recess and the horizontal distance (white dashed line) from the center of the IOF (arrows) to the plane passing through the priform aperture (arrowheads). (B) An oblique/axial image showing bilateral lateroantral canals (arrows) which are identified coursing laterally to the maxillary sinuses (asterisks) and opened by infraorbital foramina (curved arrows).

The morphometric measurements that were shown in Table 1 were performed between six reference points which are modified from the Hwang et al.^5^ and Przygocka et al.^6^ study. Those reference points:

C, the anterior margin of the IOG.
S, the posterior margin of the IOG (inferior orbital fissure).
A, the internal angulation point of the IOC.
IOF, the midpoint of the IOF.
IOR, the inferior margin of the orbital rim.
PA, the most lateral edge of the piriform aperture (PA).

**Table 1:** Definitions of measurements of the infraorbital canal.

Measurements		Definitions
IOC1 length	(A–IOF)	The distance from the internal angulation point of the infraorbital canal to the infraorbital foramen
IOC2 length	(A–C)	The distance from the internal angulation point of the infraorbital canal to the anterior border of the infraorbital groove
Total IOC length	(C–IOF)	The distance from the anterior border of the infraorbital groove to the infraorbital foramen
IOG length	(C–S)	The distance between the anterior and the posterior border of the infraorbital groove
IOF location	Superior (IOR–IOF)	The distance from the inferior orbital rim to the infraorbital foramen
	Medial (PA–IOF)	The distance from the piriform aperture to the infraorbital foramen
IOC internal angulation	Sagittal	The internal angulation of the axis of the infraorbital canal in sagittal section
	Axial	The internal angulation of the axis of the infraorbital canal in axial section
IOF entry angle	Sagittal	The angle between the axis of the infraorbital canal and the horizontal plane (Frankfort plane) that was parallel to the nasal floor
	Axial	The angle between the axis of the infraorbital canal and the vertical plane that was parallel to the sagittal plane

**Figure 5:**
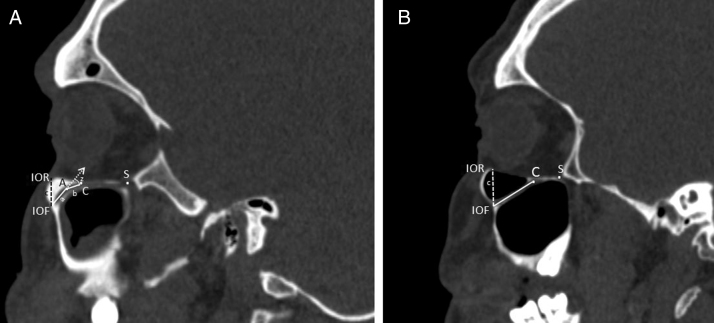
Measurements regarding to infraorbital canal, foramen and groove. A, Right parasagittal image showing the infraorbital canal with an internal angulation. The length of the IOC1 (a) was the distance from the center of the infraorbital foramen (IOF) to the internal angulation point (A). The length of the IOC2 (b) was the distance from the internal angulation point (A) to the anterior margin of the infraorbital groove (C). The length of the IOG was the distance from the anterior (C) to posterior margin of the infraorbital groove (S). The measurements of the internal angulation of the IOC (*z*) and the distance (*c*) from the infraorbital rim (IOR) to the center of the infraorbital foramen (IOF). B, Right parasagittal image showing infraorbital canal without internal angulation. The measurements of the length of the infraorbital canal (*a* + *b*) and the distance (*c*) from the infraorbital rim (IOR) to the center of the infraorbital foramen (IOF). The anterior (C) and posterior margin of the infraorbital groove (S) were identified.

We defined the IOC as the canal which is covered by bone and ending at the anterior margin of the IOG which is not covered by the bone. The internal angulation point of the IOC was identified as the change in direction of the axis of the initial IOC. After the measurements in sagittal sections were finished the horizontal plane was rotated to an oblique position that passed along the IOC. All morphometric measurements were performed in the axial section of this position. We used the piriform aperture (PA) and IOR as a reference points to identify the location of the IOF and measured the distance from IOF to PA (Fig. 4A) and IOR (Fig. 5A and B). We evaluated comprehensively the internal angulation of the IOC and whether it was absent or present (Fig. 5A and B). In IOCs with an internal angulation the length of the IOC was divided into IOC1 (initial segment) and IOC2 and were measured separately in sagittal and axial sections (Fig. 5A). The length of the IOG was measured only in sagittal sections. Also, we measured the length of the IOC and the IOG in IOCs without internal angulation (Fig. 5B).

**Figure 6:**
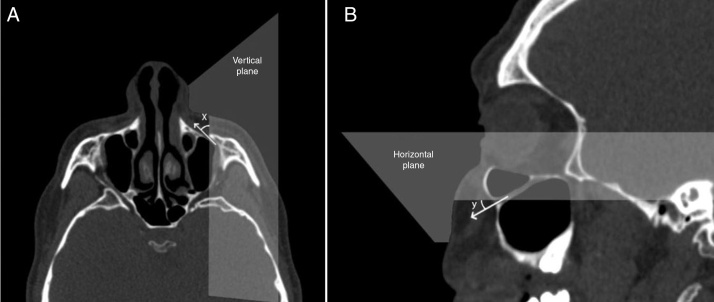
Angular measurements of the entry into infraorbital foramen in axial and sagittal sections. (A) The angle (*x*) was the angle of the axis of the infraorbital canal relative to the vertical plane that passed through the center of the infraorbital foramen. (B) The angle (*y*) was the angle of the axis of the IOC relative to horizontal plane that passed through the center of the IOF.

To identify the direction of the injecting needle we measured the angle of the axis of the IOC relative to the vertical plane which was parallel to sagittal plane, and passed through the center of the IOF in axial sections and identified it as the axial IOF entry angle (Fig. 6A). Also, the angle of the axis of the IOC relative to the horizontal plane (Frankfort horizontal plane) which was parallel to the nasal floor and passed through the IOF in sagittal sections was measured and described as the sagittal IOF entry angle (Fig. 6B). On the other hand, we analyzed the presence of the Haller cell and the maxillary sinus septa in 400 IOC's CT scans. Then, we demonstrated the relationship between the IOC corpus types and the morphometric measurements.

Statistical software SPSS 22 was used for statistical analysis. The mean, standard deviation, minimum, and maximum for each of measurements were calculated. For statistical comparisons unpaired *t*-test, Chi-square analyses and ANOVA were used and *p* < 0.05 was considered to be statistically significant.

## Results

The CT scans of patients consisted of 88 females (44%) and 112 males (56%) with a median age of 44.15 ± 17.46 years for females and 38.89 ± 17.33 years for males.

### IOC variations

Four variations of 400 IOC corpus anatomy according to relation with maxillary sinus were found as follows; Type 1 which was located within maxillary bony roof (55.3%, 221/400), Type 2 which was partially protruded into maxillary sinus (26.7%, 107/400), Type 3 which was totally protruded into maxillary sinus with a stalk (9.5%, 38/400), Type 4 which was located at the outer limit of the zygomatic recess (8.5%, 34/400).

### IOC internal angulation measurements

We determined that the axis of the IOC showed a mean internal angulation (min. 5.92° and max. 53.71°) as 28.18° ± 9.2° in sagittal sections (62.3%) and as 28.4° ± 10.0° (60%) in axial sections, respectively. Also, the prevalence of the IOCs without an internal angulation was found as 37.7% in sagittal sections and 40% in axial sections, respectively. In Table 2, we demonstrated that the IOC Type 3 mostly had no an internal angulation in both of sagittal (68.4%) and axial (63.2%) sections. Conversely, IOC Type 4 showed the highest prevalence of the internal angulation in both of sagittal (64.7%) and axial (79.4%) sections (*p* < 0.01, *p* = 0.04).

**Table 2:** The internal angulation of the infraorbital canal in relation to infraorbital canal corpus types.

Degree of the internal angulation of the IOC	Section	IOC type				*p*-Value^a^
		Type 1 No. (%)	Type 2 No. (%)	Type 3 No. (%)	Type 4 No. (%)	
IOC without angulation	Sagittal	68 (30.8%)	45 (42.1%)	26 (68.4%)	12 (35.3%)	<0.01
IOC with angulation		153 (69.2%)	62 (57.9%)	12 (31.6%)	22 (64.7%)	
IOC without angulation	Axial	98 (44.3%)	45 (42.1%)	24 (63.2%)	7 (20.6%)	0.04
IOC with angulation		123 (55.7%)	62 (57.9%)	14 (36.8%)	27 (79.4%)	

IOC, infraorbital canal; Type 1, within maxillary bony roof; Type 2, partially protruding into maxillary sinus; Type 3, totally protruding into maxillary sinus with a stalk; Type 4, located external to the zygomatic recess of the maxillary bone (lateroantral).

a Chi-square test.

### IOC and IOG lengths

In IOCs with an internal angulation the mean length of the IOC1 (initial IOC) was measured as 7.0 ± 2.7 mm and 8.0 ± 2.7 mm in sagittal and axial sections, respectively. Also, we measured the mean length of the total IOC and the IOG as 10.4 ± 2.5 mm and 18.9 ± 4.2 mm in sagittal sections, respectively as seen in Table 3. We compared the morphometric measurements with respect to gender and sides. In Table 3, we demonstrated that all morphometric measurements were higher in males than females and show statistically significant difference except for the mean axial IOF entry angle and IOC2 length (*p* < 0.05). Also, there was no statistically significant difference between sides. On the other hand, we reported that the mean length of the total IOC and the IOG was 10.6 ± 2.8 mm and 18.8 ± 4.1 mm in all IOCs and the length of the total IOC was found as the longest in IOC Type 3 and the smallest in Type 1: however, the opposite was true for the length of the IOG as shown in Table 4 (*p* < 0.001).

**Table 3:** The distribution of the comparison in morphometric measurements between females and males.

Measurements	Total	Female	Male	*p*-Value^a^
	Mean ± SD	Mean ± SD	Mean ± SD	
Sagittal IOC1 length (mm)	7.0 ± 2.7	6.7 ± 2.7	7.3 ± 2.7	0.019
Sagittal IOC2 length (mm)	3.4 ± 2.2	3.3 ± 2.1	3.5 ± 2.4	0.571
Sagittal IOG length (mm)	18.9 ± 4.2	18.1 ± 3.8	19.5 ± 4.4	<0.001
Sagittal IOF entry angle (°)	36.57 ± 8.21	34.69 ± 7.98	38.06 ± 8.51	0.014
Axial IOC1 length (mm)	8.0 ± 2.7	7.6 ± 2.6	8.4 ± 2.7	0.004
Axial IOC2 length (mm)	2.7 ± 2.8	2.4 ± 2.8	2.9 ± 2.9	0.125
Axial IOF entry angle (°)	56.80 ± 13.60	56.51 ± 13.54	56.90 ± 13.66	0.642
IOF-IOR distance (mm)	8.2 ± 1.7	7.8 ± 1.6	8.5 ± 1.7	<0.001
IOF-PA distance (mm)	13.8 ± 2.6	13.3 ± 2.6	14.1 ± 2.5	0.002

IOC, infraorbital canal; IOG, infraorbital groove; IOF, infraorbital foramen; IOR, infraorbital rim; PA, piriform aperture.

a Unpaired *t*-test. Total (*n* = 400); female (*n* = 88); male (*n* = 112).

**Table 4:** The relationship between the morphometric measurements and the infraorbital canal corpus types.

Measurements	IOC type				*p*-Value^a^
	Type 1	Type 2	Type 3	Type 4	
	Mean ± SD	Mean ± SD	Mean ± SD	Mean ± SD	
(S) Total IOC length (mm)	9.6 ± 2.1	10.8 ± 2.2	14.9 ± 3.4	10.8 ± 2.3	<0.001
(S) IOG length (mm)	19.4 ± 3.8	18.9 ± 3.9	14.7 ± 5.1	18.5 ± 4.3	<0.001
(S) IOF entry angle (°)	38.13 ± 11.67	36.84 ± 8.02	30.67 ± 7.47	32.22 ± 7.95	0.003
(A) Total IOC length (mm)	9.8 ± 2.0	11.0 ± 2.0	15.0 ± 2.5	11.4 ± 2.3	<0.001
(A) IOF entry angle (°)	57.66 ± 13.64	55.36 ± 13.46	54.20 ± 12.17	57.81 ± 13.85	0.018
IOF–IOR distance (mm)	7.7 ± 1.3	8.3 ± 1.7	10.5 ± 1.7	7.3 ± 1.5	<0.001
IOF–PA distance (mm)	13.7 ± 2.6	13.7 ± 2.6	13.9 ± 2.1	14.0 ± 2.7	0.925

S, sagittal; A, axial; IOC, infraorbital canal; IOG, infraorbital groove; IOF, infraorbital foramen; IOR, infraorbital rim; PA, piriform aperture; Type 1, within maxillary bony roof; Type 2, partially protruding into maxillary sinus; Type 3, totally protruding into maxillary sinus with a stalk; Type 4, located external to the zygomatic recess of the maxillary bone (lateroantral).

a ANOVA.

## IOF location and entry angles

The mean distances between the IOF-PA and IOF-IOR were found as 13.8 ± 2.6 mm and 8.2 ± 1.7 mm, respectively. The distance from the IOF to IOR in IOC Type 3 was quite longer than that in other types, but the opposite was true for the IOC Type 1 and 4 as shown in Table 4 (*p* < 0.001). Conversely, the IOF-PA distance did not show a statistically significant difference with respect to the IOC corpus types (*p* = 0.925). We measured the mean sagittal IOF entry angle as 36.57° ± 8.21° and axial IOF entry angle as 56.80° ± 13.60°, respectively. We reported that sagittal and axial IOF entry angle values were smaller in IOC Type 3 and larger in Type 1 than that in other types in Table 4 (*p* = 0.003, *p* = 0.018). In Table 5, the prevalence of the presence of the Haller cell and the maxillary sinus septa were indicated and the correlation of them with specific IOC types was not found statistically significant (*p* = 0.06, *p* = 0.145).

**Table 5:** The surrounding anatomical structures in maxillary sinus in relation to the infraorbital canal corpus types.

Surrounding structures	IOC type				*p*-Value^a^
	Type 1	Type 2	Type 3	Type 4	
No maxillary septa	176 (79.7%)	65 (60.8%)	25 (65.8%)	22 (64.8%)	0.060
>0 maxillary septa	45 (20.3%)	42 (39.2%)	13 (34.2%)	12 (35.2%)	
No Haller cell	193 (87.4%)	8 (79.7%)	28 (73.7%)	29 (85.3%)	0.145
>0 Haller cell	28 (12.6%)	21 (19.6%)	10 (26.3%)	5 (14.7%)	

IOC, infraorbital canal; Type 1, within maxillary corpus; Type 2, partially protruding into maxillary sinus; Type 3, totally protruding into maxillary sinus; Type 4, located external to the zygomatic recesses of the maxillary bone (lateroantral).

^a^ Chi-square test.

## Discussion

Traumatic ION injury especially in zygomaticomaxillary complex fractures, often results in numbness of the midface and ipsilateral paresthesia. The occurrence ratio of the paresthesia and permanent sensory disturbance in innervation area of the ION was 30–80% in patients with maxillary fractures.^7^, ^8^ The ION is well protected by the bony roof of the maxillary sinus, but increasing degree of the protrusion of the IOC into the maxillary sinus can be associated with canal dehiscence and thinner bony sheet. So, iatrogenic ION injury has been reported during interventional procedures like periorbital endoscopic approaches, orbital reconstructive surgery, Caldwell-Luc operation, regional block anesthesia and radiofrequency neurotomy of trigeminal ganglion.^1^, ^2^, ^9^ The incidence of the iatrogenic temporary ION hypoesthesia was reported as 0.5% during midfacial lift surgery. As a result, postoperative trigeminal neuropathy, corneal anesthesia with keratitis, retrobulbar hematoma and neurovascular injury of the IOC can ocur.^3^ The anatomic knowledge of the location of the IOC with its surrounding structures and preoperative CT imaging of the IOC corpus types is important and guides the surgeon to the most appropriate approach which will avoid iatrogenic ION injury.^10^, ^11^

Yenigun et al. classified the configuration of the IOC into three types and reported that the most common type was Type 2 (51.2%).^11^ Ference et al. indicated that the IONs were categorized as Type 1 (60.5%), Type 2 (27.0%), Type 3 (12.5%).^2^ Lantos et al. evaluated retrospective 500 CT scans and reported that protrusion of the IOC into maxillary sinus was identified in 10.8% of patients (5.6% bilateral, 5.2% unilateral).^1^ In our study, we consider lateroantral type as named IOC Type 4 that was initiated defined in the study by Rusu et al. We reported that the prevalence of the IOC corpus types as Type 1, 55.3%; Type 2, 26.7%; Type 3, 9.5%; Type 4 (lateroantral), 8.5%. In particular, the IOC Type 2 and Type 3 with increasing degree of the protrusion of the IOC seems the most likely to be exposed to injury during surgical procedures such as endoscopic sinus surgery, maxillary sinus operations and reconstruction of the orbital floor. During a Caldwell-Luc approach and extended endoscopic sinus surgery to excise mucosal disease or tumor from the roof of the maxillary sinus, the periosteum of the orbital floor was dissected carefully to avoid iatrogenic ION injury in the IOC Type 2 and Type 3.^1^, ^3^ The lateroantral type that can determine modifications of common procedures should be kept in mind by dental surgeons preoperatively.^4^

Previous anatomical studies dealing with the morphometry of the IOC, IOF and IOG have been performed by using CT scans, skulls, cadavers and photogrammetry.^1^, ^5^, ^6^, ^10^, ^12^, ^13^, ^14^ Especially, the cadaveric-dried skulls were mostly used and showed limitations related to convenience in living actual patients and imaginary sections. The craniofacial bones can be evaluated from various angles by using CT.^5^ As a result of the proper application of the multiple planar reconstructions technique the length, angulation of the IOC and the location of the IOF can be determined accurately. In literature, the compact study which investigating whether IOC morphometric parameters are altered by the IOC corpus types not found. In this respect, this study has a new methodology analysing the relationship between them. In this study, we carried out an in depth study about the internal angulation of the IOC. As a result, we reported that there were an internal angulation of the IOC in sagittal and axial sections as a mean 28.18° ± 9.2° and 28.4° ± 10.0°, respectively. Also, the prevalence of the internal angulation of the IOC was found in a very high ratio in both sagittal and axial sections (62.3% and 60%). Our study also found an association between the internal angulation and the IOC corpus types. As seen in Table 2, IOC Type 4 mostly shows an internal angulation in both of sagittal (64.7%) and axial (79.4%) sections. Conversely, there was commonly no internal angulation in the IOC Type 3 in both of sagittal (68.4%) and axial (63.2%) sections (*p* < 0.01, *p* = 0.04) and also the length of the IOC varied from 8 to 20 mm.

In IOCs with an internal angulation to indicate the depth of the puncture we measured the mean length of the initial IOC (IOC1) as 7.0 ± 2.7 mm in sagittal and 8.0 ± 2.7 mm in axial sections and demonstrated the length of the IOC1 > IOC2 in both of sagittal and axial sections in Table 3. The internal angulation decreases the depth of the puncture by decreasing the length of initial IOC and complicates the ION block and radiofrequency neurotomy. In the literature, the measured soft tissue thickness over the IOF ranged from 12 to 19 mm. So, the depth of the puncture can be ranged between 17 and 25 mm during radiofrequency neurotomy, with the needle being inserted not more than 5 mm into the IOC to avoid orbital injury and skin ulceration.^5^, ^15^

In previous studies using dried skulls, the measured mean lengths of the IOC ranged from 12.75 to 23 mm and IOG length values ranged as 6–16 mm.^3^, ^6^, ^15^, ^16^ Using CT scans Hwang et al. reported that the mean length of the IOC, IOG and total IOC/IOG complex were measured as 11.7 mm, 16.7 mm and 28.4 mm.^5^ In this study, we reported that the mean length of the total IOC and the IOG was 10.6 ± 2.8 mm and 18.8 ± 4.1 mm in sagittal sections of all IOCs. So, the mean total length of the IOC and IOG was measured as 29.5 mm that was similar to results in CT studies. In addition, the length of the total IOC was found as the longest in IOC Type 3 and the smallest in Type 1 but, the opposite was true for the length of the IOG as seen in Table 4 (*p* < 0.001).

On the other hand, to identify the direction of the needle the angles of the axis of the IOC relative to vertical and horizontal planes are measured in previous studies.^5^, ^12^, ^13^ We reported that the mean sagittal and axial IOF entry angles were measured as 36.57° ± 8.21° and 56.80° ± 13.60°, respectively. These measurements showed consistency but, still larger than that in other studies due to different definitions of angles. According to Table 4 the sagittal and axial IOF entry angle values were significantly smaller in IOC Type 3 but, larger in IOC Type 1 than that in other types (*p* = 0.003, *p* = 0.018). As a result, the IOC Type 3 with no internal angulation, the longer length of the IOC and the smaller IOF entry angle can facilitate the ION block and radiofrequency neurotomy but complicate surgical operations.

Most studies using dried skulls reported that the IOF-IOR and the IOF-PA distances ranged as 5–10.9 mm and 14–18 mm, respectively.^3^, ^6^, ^12^, ^13^, ^16^, ^17^, ^18^ Also, the studies using CT scanning reported that the measured mean distances between IOR and IOF ranged between 9.04 and 10 mm.^5^, ^14^ We measured the mean distances from IOF to IOR and PA as 8.2 ± 1.7 mm and 13.8 ± 2.6 mm similar to them. Notably, we analyzed that the IOF-IOR distance was significantly longer in IOC Type 3 than that in other types (*p* < 0.001) but, the IOF-PA distance had no statistically significant relationship with the IOC types (*p* = 0.925) as shown in Table 4. So, the IOF-IOR distance increased parallel to the degree of the protrusion of the IOC which may cause the iatrogenic ION injury during surgical interventions.

Cakur et al. and Yenigun et al. and Koymen et al. reported that the prevalence of the maxillary sinus septa was 35.3% and 16.5%, respectively.^11^, ^19^, ^20^ In our study, we determined the prevalence of the presence of the maxillary sinus septa as 28% (40.2% with Type 1; 37.5% with Type 2; 11.6% with Type 3; 10.7% with Type 4) in Table 5. But, a statistically significant correlation with the IOC corpus types was not found (*p* = 0.06). The prevalence of the presence of the Haller cell was 16% and there was no a statistically significant correlation between the presence of Haller cell and IOC types (*p* = 0.145).

## Conclusion

These results again emphasized the value of preoperative CT imaging, which can offer accurate understanding of the regional anatomy of and around the IOC. We take into account that there were personal variations in the IOC corpus types which affect the localization of the IOF and the morphometric parameters. Therefore, radiologic identification of the specific localization for each corpus type will play a key role when choosing an appropriate surgical approach to avoid iatrogenic ION injury and help to surgeon when performing anesthetic interventions. Also, the success rate of the maxillofacial surgery and regional block anesthesia can be increased.

## Conflicts of interest

The authors declare no conflicts of interest.
